# Adapting to a new normal: Antiracism as a core public health principle

**DOI:** 10.1371/journal.pgph.0000037

**Published:** 2021-10-13

**Authors:** Utibe R. Essien, Eloho O. Ufomata

**Affiliations:** 1 Center for Health Equity Research and Promotion, VA Pittsburgh Healthcare System, Pittsburgh, PA, United States of America; 2 Division of General Internal Medicine, University of Pittsburgh School of Medicine, Pittsburgh, PA, United States of America; PLOS Global Public Health, UNITED STATES

“We are now faced with the fact that tomorrow is today. We are confronted with the fierce urgency of now. In this unfolding conundrum of life and history there is such a thing as being too late…This may well be mankind’s last chance to choose between chaos and community.”–Dr. Martin Luther King, Jr., 1967

As COVID-19 vaccination slowly upticks around the world and we are faced with the new challenge of the Delta variant of the novel coronavirus, our global collective is once again reminded of the impacts of inequity in resource distribution and faced with a moment of critical reflection. An opportunity to examine the past 18 months, and ask ourselves, where do we go from here? Will we go back to “normal,” as so many have hoped for in the past year and a half? A normal that resulted in Black, Hispanic, and Native Americans suffering 2–2.5 times the rate of infection and death during the pandemic [1]. A normal that has resulted in 70% of individuals in the U.S. receiving at least one COVID-19 vaccine by August 2021, a time point when fewer than 1% of Africa’s 3 billion people were vaccinated [2]. In the United States, we saw Black and Hispanic people receive smaller shares of vaccines compared to the disproportionate impact of the disease on their communities and also compared to their nationally representative population [3]. A normal created by centuries of racism and a lack of acknowledgement of its impact as the driver of disparities in disease outcomes [4].

Over the past year, spurred by the grass-roots movement of Black people across the world demanding that our lives matter, we have experienced a growing fervor to acknowledge and abolish racism and disparities in care from our health care institutions. Antiracism trainings have been included in medical school curricula across North America and Europe, as students and educators have called out this critical gap in health professional training [5]. Health care advocates have sought to rid clinical care of the pervasive legacy of the biologic determinism of race and rather encouraged all care providers to understand that race is a sociopolitical construct [6]. Researchers have drawn attention to the sordid history of racism and discrimination in public health and medical research and have made a commitment to look beyond mistrust as the driver of underrepresentation of diverse communities within their scientific endeavors [7]. Yet, with all these steps, the question remains: can this work be sustainable? The answer, we maintain, must be a resounding yes, and we offer a number of strategies to achieve this goal.

First, the health system must commit to desegregation at every level. While black and white photographs of “Whites Only” clinic waiting rooms remind us of a chilling past in the U.S., the legacy of segregation and white supremacy persists in our health system today. Prior research has found that Black and Latinx patients are more likely to be cared for by a resident physician in training than white patients [8]. Another study observed that Black patients with heart failure were less likely than White patients to be admitted to specialized cardiology units in the hospital, despite similar levels of care needs [9]. These examples are pervasive across the globe, with Black and brown communities consistently being relegated to the lowest levels of medical care, either by insurance status in the U.S.; or by lack of access to lifesaving resources in economically developing countries, such as has been seen in several nations in the Global South with the latest COVID-19 surge [10–12]. In addition, there has been growing concern for how the requirement of proof of vaccination to access resources in certain communities might disproportionately impact communities of color who have not had equal access to vaccinations. To sustain antiracism in medicine, we must ensure that regardless of race, or economic status, all patients can have access to the highest quality of care.

Second, we must divest from racist practice and policy. The health system, like any other institution, functions according to design. And so, we must consider how access to medical care disadvantages segments of our population. The potentially triggering effect experienced by patients of color, when no one on the medical team looks like them, or speaks their language. Or the impossibility of showing up on time for a 9AM-5PM outpatient clinic appointment for individuals with limited job security, financial assets, and social support. Or on a global scale, encouraging capitalism as the driver for scientific discovery. These seemingly colorblind policies, including the physical accessibility of certain health care settings due to centuries of discriminatory U.S. housing and public transportation policy, are just some of the areas we can intervene upon to ensure an antiracist public health system [13].

Third, we must diversify the health professions workforce. For example, in the U.S, the rates of racial and ethnic minorities in medicine are dismal, with recent data reporting 4.9% of all medical school matriculants identified as Black and Hispanic with even fewer identifying as Native American [14]. Myriad data demonstrate the importance of patient-provider racial, including higher likelihood of influenza vaccination or acceptance of an invasive cardiovascular procedure [15]. The COVID-19 pandemic has especially brought the lack of diversity of the public health workforce to the forefront, highlighting the need for trusted messengers that not only look like the community, but can speak their language and empathize with their lived experience. Sustaining antiracism in public health will require that we no longer center traditional health care voices but rather amplify those who can boldly speak to the pervasive role racism has on all aspects of our health.

Fourth, we have to ensure a systemic approach to antiracist public health training for health professionals, particularly clinicians. There is a notable paucity of published medical school curricula that teach antiracism [16]. As such, health professional students continue to train in an ahistorical environment, with limited understanding of how racism influences health care around the world. Such an environment results in dangerous false beliefs about biological differences between Black and White patients, for example, as well as critical gaps in the training of a future generation of health system leaders [17]. We must push forth an antiracist educational agenda, across all healthcare professional training across the globe, and urge all licensing institutions to adopt measurable competencies and targets that will sustain these efforts.

Finally, we must deepen our investments in the community. As the pandemic has revealed, the social determinants of health, and a focus on public health more specifically, are critical to the wellbeing of each member of our global society. Prior research in Massachusetts, U.S, found that a 10% increase in the Hispanic or Black population was associated with an increase of 250–315 COVID-19 cases per 100,000 population [18]. These higher rates were not associated with biologic differences between the races but rather differential pollution exposure, household size, and rates of essential workers. We must advance public health and policy efforts to improve care for traditionally underserved communities while encouraging health system leaders to embrace their role as anchor institutions that provide social supports such as food, housing, and employment assistance to those in need [19].

It is our great desire for a post pandemic world, one in which we can once again remove the mask and walk through the world as our full selves. However, we cannot fully experience a post COVID-19 world without correcting the deep fault lines within our health system that have been unmasked in the past year and a half. With the mask off, we can clearly envision a better future; one that understands that racism is a public health issue and one where we are all actively working together to be antiracist in pursuit of a just and equitable health system for all.
